# Urban environments and objectively-assessed physical activity and sedentary time in older Belgian and Chinese community dwellers: potential pathways of influence and the moderating role of physical function

**DOI:** 10.1186/s12966-020-00979-8

**Published:** 2020-06-09

**Authors:** Ester Cerin, Delfien Van Dyck, Casper J. P. Zhang, Jelle Van Cauwenberg, Poh-chin Lai, Anthony Barnett

**Affiliations:** 1 Mary MacKillop Institute for Health Research, Australian Catholic University, Melbourne, Victoria Australia; 2 School of Public Health, The University of Hong Kong, Hong Kong, Hong Kong SAR China; 3 Baker IDI Heart and Diabetes Institute, Melbourne, Victoria Australia; 4 Department of Movement and Sport Sciences, Faculty of Medicine and Health Sciences, Ghent University, Ghent, Belgium; 5 Research Foundation Flanders (FWO), Brussels, Belgium; 6 Department of Public Health and Primary Care, Faculty of Medicine and Health Sciences, Ghent University, Ghent, Belgium; 7 Department of Geography, Faculty of Social Sciences, The University of Hong Kong, Hong Kong, Hong Kong SAR China

**Keywords:** Walkability, Accelerometry, Older adults, Europe, China, Neighbourhood environment, Sedentary time, Moderate-to-vigorous physical activity, Physical function

## Abstract

**Background:**

Many studies have examined neighbourhood environmental correlates of older adults’ physical activity (PA) but only a few focused on sedentary time (ST). Only a small proportion of these studies used objective measures of PA/ST, such as accelerometer-assessed PA/ST, and only a couple employed accelerometer cut-points appropriate for older adults. Furthermore, although older adults experience declines in physical function as they age, there is a dearth of information on the impact of the neighbourhood environment on PA/ST in individuals with different levels of physical function.

**Methods:**

We used data from two extant cross-sectional studies conducted in Hong Kong (China) and Ghent (Belgium) (*N* = 829). Participants were recruited from pre-selected administrative units stratified by socio-economic status and walkability. Moderate-to-vigorous PA (MVPA) and ST were assessed for 7 days using accelerometers and cut-points developed for older adults. Objective neighbourhood environmental attributes within 400 m and 1 km buffers surrounding participants’ homes were quantified using Geographic Information Systems data. Lower extremity physical function was objectively assessed. Socio-demographic information was collected via interviews. Total, direct and indirect (mediated) effects of environmental attributes on MVPA and ST were estimated using generalised additive mixed models and the joint-significant test.

**Results:**

Commercial/civic destination density and number of parks within 1 km from home showed positive total and direct effects on MVPA, and public transport density showed negative total and direct effects on ST, which were consistent across cities and physical function levels. The total and direct effects of residential density on MVPA depended on physical function, and those of residential density on ST differed by city. A complex network of potential inconsistent pathways linking all environmental attributes to MVPA and ST in the whole sample or in subgroups of participants was revealed.

**Discussion:**

Access to parks and commercial/civic destinations appear to support older adults’ MVPA in different geographical and cultural contexts and irrespective of their physical function level. By supporting MVPA, these characteristics also contribute to a reduction in ST. The potential effects of public transport, recreational facilities and residential density are less straightforward and point at inconsistent effects that may depend on the geographical context and level of physical function.

## Background

A plethora of evidence suggests that regular engagement in moderate-intensity physical activity (PA) confers numerous health benefits to older adults, such as lower risk of cardiovascular disease, diabetes, cognitive decline and some types of cancer [1, 2]. A smaller body of research also suggests that sedentary time (ST) - defined as any waking behaviour characterized by an energy expenditure ≤1.5 METs (Metabolic Equivalents of Task) while in a sitting, reclining or lying posture [3] - is positively associated with all-cause mortality, lower cognitive function and a range of chronic conditions [4, 5].

To promote PA and reduce ST among ageing populations, it is important to create activity-friendly neighbourhoods [6, 7]. The characteristics of proximal neighbourhood environments are especially relevant to older adults who, due to declines in mobility, are particularly adversely impacted by environmental factors that discourage an active lifestyle [8]. Many studies have examined neighbourhood environmental correlates of PA in older adults [9, 10] whilst only a few have focused on sedentary behaviour [11–13]. In general, these studies indicate that living in neighbourhoods with good vs. poor access to commercial destinations, public transport, parks and recreational facilities is associated with higher levels of PA [9, 10] and somewhat less ST [11, 12].

Several shortcomings of published research on environmental correlates of older adults’ PA, that also apply to ST, have been recently highlighted [9]. First, the great majority of studies employed self-report measures of PA and ST [9, 11] which may yield culturally-biased responses [14] and provide less accurate estimates of activity levels than their objective counterparts (e.g., accelerometers) [15]. The latter holds especially for older populations who are more likely to participate in lower-intensity PA and accumulate more ST, which can be difficult to report accurately [16]. Second, most studies that examined accelerometer-assessed moderate-to-vigorous PA (MVPA) and ST employed cut-points that were developed for younger populations and, hence, likely underestimated the time that older adults spent in MVPA [9, 17] and overestimated ST [18]. Optimally, the estimation of older adults’ MVPA via accelerometers should be based on cut-points appropriate for the age group such as those established by Barnett and colleagues [17] or Copeland and Esliger [19]. Third, there is a dearth of information on the moderating effects of lower extremity physical function on the environment-MVPA/ST relationships [9]. Balance difficulties and declining muscle strength with ageing result in changes in gait patterns and efficiency [20] that, in turn, increase the inter-individual variability in the relationship between accelerometer counts and walking speed [17]. Examining the moderating role of physical function is also important from a substantive viewpoint. In fact, there is some evidence that access to various destinations and amenities may have a greater impact on PA among older adults with mobility problems [12, 21–23]. However, only one of these studies employed accelerometers to measure PA [12] and none used accelerometer cut-points appropriate for older adults.

Limitations in the study design and analytical approaches have also been reported [9]. As the effect of the environment on PA is usually modest, to accurately characterize the shape of dose-response relationships, it is important to ensure a sufficient amount of variability in environmental exposures [24]. Several studies have achieved this by sampling from neighbourhoods with different environmental characteristics [12, 25–28]. However, most of these studies were conducted in single locations with limited variability in the built environment, while multi-country studies in adults [29–32] suggest that the pooling of comparable data from environmentally diverse cities and countries is needed to address the issues arising from insufficient variability in exposures.

Furthermore, previous studies employed analytical approaches that did not consider the potential causal pathways that link environmental characteristics with PA and ST, resulting in potentially biased or misinterpreted estimates of environmental-PA/ST associations [33]. For example, whilst residential density is a plausible precursor and, thus, a potential confounder of the effects of access to amenities and public transport on PA [34], most studies have failed to adjust for residential density [9]. Also, studies that mutually-adjusted for the effects of residential, public transport and/or amenity density on PA misinterpreted the regression coefficient of residential density as representing its total independent effect on PA, whilst this held true only for the regression coefficients of public transport and/or amenity density [35, 36]. An examination of potential causal pathways is also necessary to understand how specific environmental characteristics impact on older adults MVPA and ST. For example, residential density may impact on MVPA by increasing access to services and public transport that, in turn, may reduce car dependency and, thus, promote active transport [8, 11].

To address the above-mentioned limitations, this study examined the potential mediators of the relations between objectively-assessed characteristics of the neighbourhood environment and objectively-assessed MVPA and ST in older adults, and the moderating effects of lower extremity physical function. To achieve these objectives, pooled comparable data from two extant cross-sectional studies conducted in two urban locations with different levels of density – namely, Ghent (Belgium) and Hong Kong (China) – were used.

## Methods

The Active Lifestyle and the Environment in Chinese Seniors (ALECS) [37] and Belgian Environmental Physical Activity Study on Seniors (BEPAS Seniors) [38] studies are observational epidemiologic cross-sectional studies conducted, respectively, in Hong Kong, China (2012–2016) and Ghent, Belgium (2010–2012). They both aimed to examine the relationships of neighbourhood environmental characteristics with physical activity, sedentary behaviours and health outcomes in older community dwellers and used comparable study design and measures. This study used information collected on ALECS (*n* = 402) and BEPAS (*n* = 427) participants who provided valid accelerometer data on PA and ST (total *N* = 829).

### Participants

Details about participant recruitment and selection for the ALECS [12, 21, 37] and BEPAS [38] studies have been described previously. Briefly, both studies employed a two-stage sampling strategy whereby participants were evenly recruited from pre-selected census administrative units (neighbourhoods) stratified by socioeconomic status (SES; median household income) and transport-related walkability (a composite measure of dwelling density, street intersection density and land use mix) to maximise variability in environmental exposures (e.g., dwelling density, access to services, etc). Hong Kong participants were selected from 124 Tertiary Planning Units (TPUs), the smallest administrative units with publicly-available census-level data [37]. Ghent participants were selected from 20 neighbourhoods, each consisting of 1 to 5 adjacent statistical sectors, the smallest census administrative units for which socio-demographic data were available [38].

Due to privacy regulations restricting access to personal contact information, Hong Kong participants (≥65 years) were recruited in person from the Elderly Health Centres (EHCs) of the Department of Health (72%) and from elderly community centres in the selected neighbourhoods (28%). Eligibility criteria included being able to speak Cantonese, living in one of the selected neighbourhoods for at least six months, being able to walk unassisted for at least 10 m and being cognitively intact. Women and those living in more walkable neighbourhoods were more likely to consent participating in the study (all *p*s < .001). A total of 909 participants were enrolled in the study (71% response rate). Of these, ~ 45% (i.e., 416) were randomly selected to wear an accelerometer to objectively assess their PA and ST and 402 provided valid accelerometer data as defined in the Measures section below [37]. In Ghent, 1750 older adults (≥65 years) were randomly selected from the statistical sectors and received an information letter through mail with a follow-up home visit approximately one week after receiving the letter. Eligibility criteria included being able to speak Dutch, living independently and able to walk 200 m without severe physical restrictions. A total of 508 participants were enrolled in the study (508 out of 1128 potential participants found at home after three attempts; 45% response rate). Of these, 427 participants provided valid accelerometer data. All participants provided written informed consent. Data were collected in the participants’ homes in Ghent and in health/community centres in Hong Kong by trained interviewers. Sample characteristics are presented in Table 1.

**Table 1:** Sample characteristics

Characteristics [% missing values]	ALECS & BEPAS (n = 829)	ALECS (*n* = 402)	BEPAS (*n* = 427)
**Participant demographics and physical function**			
Age (years; mean ± SD) [0.1% missing]	74.83 ± 6.18	75.55 ± 6.15	74.16 ± 6.14
Sex (% female) [0.1% missing]	61.28	68.91	54.10
Education (% above primary school) [0.1% missing]	61.04	46.77	74.47
Marital status (% married or cohabiting) [0.1% missing]	64.66	62.94	66.28
Neighbourhood SES (low/high, % low) [0.1% missing]	48.73	49.25	48.24
Household car ownership (% yes) [0.2% missing]	55.49	28.86	80.56
SPPB (range 1–12, mean ± SD) [1.0% missing]	10.03 ± 1.94	9.94 ± 1.95	10.11 ± 1.93
**Objective neighbourhood attributes (mean ± SD) [0% missing]**			
Residential density (dwellings/km^2^)			
400 m SNR buffers	21,952 ± 58,736	37,227 ± 81,358	7569 ± 6866
1 km SNR buffers	21,546 ± 21,211	36,316 ± 21,490	7640 ± 6328
Commercial & civic destination density (destinations/km^2^)			
400 m SNR buffers	155.74 ± 140.61	185.87 ± 107.23	127.38 ± 161.07
1 km SNR buffers	119.98 ± 112.51	118.27 ± 60.33	121.58 ± 145.52
Public transport density (stops/km^2^)			
400 m SNR buffers	16.56 ± 17.38	14.86 ± 18.67	18.16 ± 15.92
1 km SNR buffers	14.10 ± 9.84	10.74 ± 6.87	17.27 ± 11.09
Recreation density (facilities/km^2^)			
400 m SNR buffers	14.65 ± 18.35	19.61 ± 23.83	9.99 ± 8.65
1 km SNR buffers	14.36 ± 13.35	22.17 ± 15.17	7.00 ± 4.23
Park number			
400 m SNR buffers	1.47 ± 1.69	1.21 ± 1.46	1.71 ± 1.85
1 km SNR buffers	7.15 ± 6.24	6.37 ± 5.12	7.88 ± 7.07
**Accelerometer-assessed physical activity and sedentary time (mean ± SD) [0% missing]**			
Moderate-to-vigorous physical activity (average min/day)	55.78 ± 37.05	69.00 ± 36.31	43.33 ± 34.24
Sedentary time (average min/day)	414.13 ± 98.01	404.94 ± 98.11	422.78 ± 97.25
Average wear time (average min/day)	835.38 ± 87.31	811.71 ± 92.22	857.66 ± 76.06

*ALECS* Active Lifestyle and the Environment in Chinese Seniors study, *BEPAS* Belgian Environmental Physical Activity Study on Seniors study, *SD* standard deviation, *SES* socio-economic status, *SPPB* Short Physical Performance Battery, *SNR* street-network residential

### Measures

#### Neighbourhood environmental characteristics

Participants’ neighbourhoods were defined as 400 m- and 1 km-radius street-network buffers surrounding a participant’s residential address. These distances are considered to be walkable as they correspond to an actual 10–20 min walk in any direction from home [12, 39]. Geographic Information Systems (GIS) data were used to quantify neighbourhood environment characteristics for each participant and each buffer size. Neighbourhood residential density (dwellings/km^2^) was computed as a measure of urban densification. Other environmental attributes that were calculated included: number of parks contained within or intersected by a residential buffer; density of food and retail outlets (outlets/km^2^); civic and institutional destinations (destinations/km^2^); entertainment destinations (destinations/km^2^); recreation density (destinations/km^2^) and public transport density (stops/km^2^). Given that densities of food, retail, entertainment, civic and institutional destinations were highly correlated in Ghent (r > 0.90), these measures were combined into a commercial and civic destination density variable (destinations/km^2^). Spatial analyses were conducted using ArcGIS (version 10.3; ESRI) software.

#### Accelerometer-assessed moderate-to-vigorous physical activity and sedentary time

MVPA and ST were objectively assessed using GT3X/GT3X+ Actigraph accelerometers (Fort Walton Beach, FL, USA), which are valid and reliable tools to assess PA and ST in older adults [19, 40]. Participants wore the accelerometer above the right hip for seven consecutive days during waking hours, except when engaging in water-based activities. Data were collected at 60-s epochs [41, 42] using the Low Frequency Extension (LFE) filter. Only vertical-axis data were analysed in the present study. Periods with ≥90 min of consecutive zero accelerometer counts were categorized as non-wear time [43]. Days with at least 10 h of recorded wear time were considered valid. A participant was included in the analysis if s/he had at least five valid days of data, including at least one weekend day. MVPA and ST were defined using accelerometer cut points developed specifically for older adults and using the LFE filter. Specifically, values < 25 cpm were categorised as ST [44] and values ≥1013 cpm were categorised as MVPA [17].

#### Covariates, mediators and moderators

Information on participants’ age, sex, educational attainment, marital status and household car ownership was collected using an interviewer-administered questionnaire. A dichotomous measure of neighbourhood-level SES (high vs. low SES) was created using census data on administrative-level median household income. All variables were treated as covariates apart from household car ownership which was treated as a mediator of environment-MVPA/ST relationships. Physical function was quantified as the total score on the Short Physical Performance Battery (SPPB) [45]. The SPPB is a validated measure of lower extremity physical function with excellent metric characteristics. The total score on the SPPB and study site (Ghent vs Hong Kong) were considered as covariates and potential moderators of environment-car ownership- MVPA/ST associations.

### Statistical analyses and hypotheses

Descriptive statistics were computed for all variables. As there were only 11 cases (1.3%) with missing data on at least one variable, no multiple imputations were planned and the analyses were conducted on complete cases (*n* = 818).

**Fig. 1:**
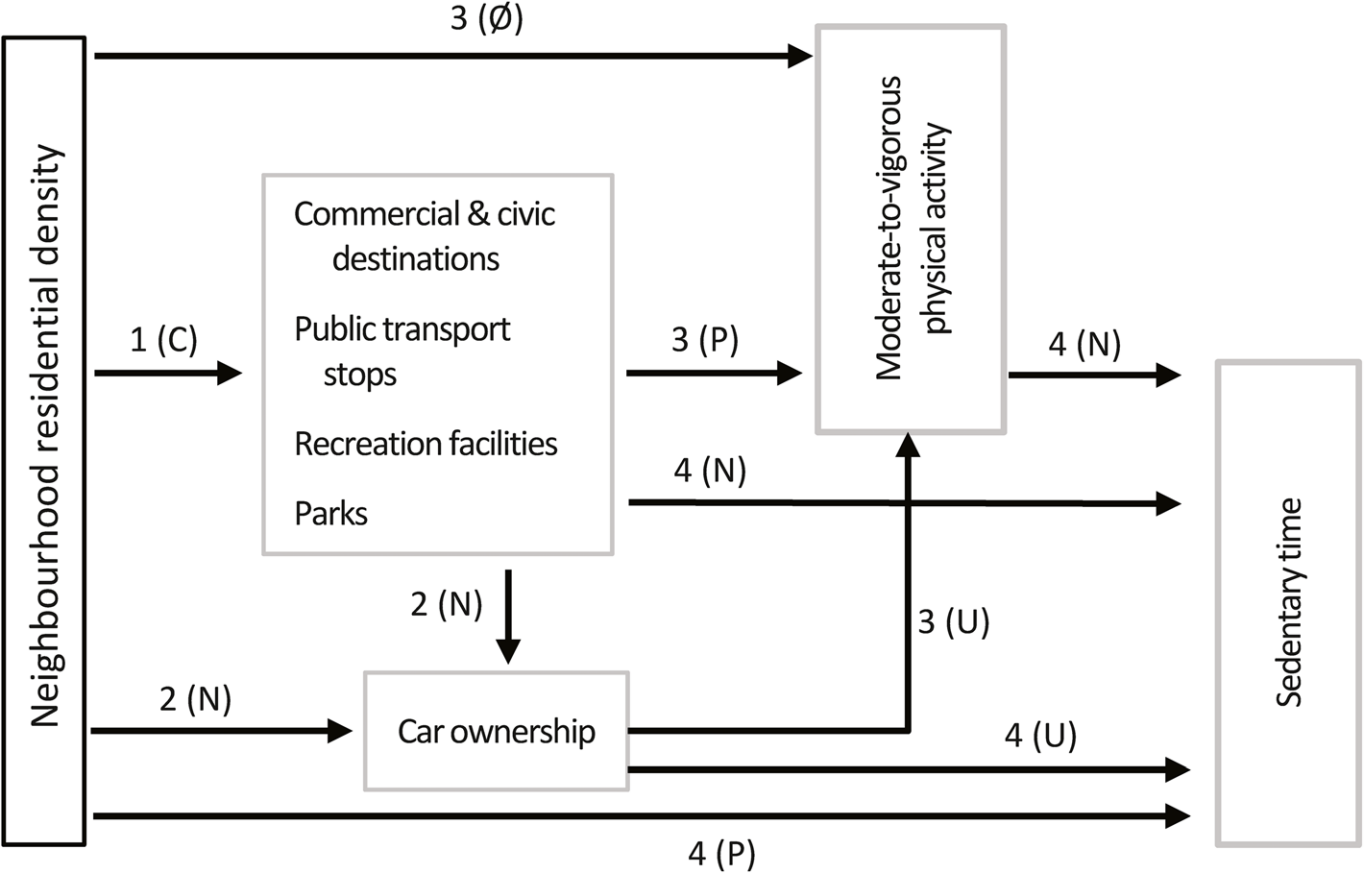
Conceptual model of associations of neighbourhood environmental characteristics with moderate-to-vigorous physical activity and sedentary time. Pathways 1 to 4 indicate associations of environmental characteristics with moderate-to-vigorous physical activity and sedentary time mediated by car ownership and moderated by city (pathways 1–4) and physical function (pathways 2–4). N, P, Ø and C indicate, respectively, hypothesised negative, positive, null and curvilinear relationships between variables. The letter U indicate uncertain direction of association

This study aimed to identify neighbourhood environment characteristics potentially affecting older adults’ MVPA and ST, mediators underlying these associations, the moderating role of physical function and the extent to which these associations were generalisable across study sites (Hong Kong and Ghent) (Fig. 1). Directed acyclic graphs were used to inform mediation analyses and the selection of a minimal sufficient set of confounders of exposure-mediator-outcome relationships (Additional file 1, Fig. S1). Exposure-mediator- outcome associations and the moderating effects of study site and SPPB were estimated using generalized additive mixed models (GAMMs) [46] accounting for dependency in error terms due to area-level clustering (participants sampled from selected administrative units). GAMMs can model curvilinear relationships of unknown form, which allow the characterisation of dose-response relationships. Separate sets of GAMMs for neighbourhood attribute measures based on 400 m and 1 km residential buffers were estimated. Curvilinear associations were estimated using smooth terms modelled with thin plate splines [46]. If the data did not provide sufficient evidence of a curvilinear association, smooth terms were replaced by linear terms. Model selection (linear vs. curvilinear effect) was based on Akaike Information Criterion (AIC) values, where a lower AIC was indicative of a better-fitting model. A ≥ 5-unit difference in AIC was used as the criterion for model selection [31, 47]. Potential multicollinearity was assessed by computing the Variance Inflation Factor (VIF) for each variable included in the models. A VIF > 5 was considered to be problematic.

#### Total effects of neighbourhood environmental attributes on MVPA and ST

Total effects refer to the mediated plus unmediated effects of an exposure (environmental variable) on the outcome (MVPA or ST). They are estimated by models that do not include potential mediators. The confounder-adjusted total effects of neighbourhood environment characteristics on MVPA and ST and the moderating roles of SPPB and study sites were first estimated. Confounders included factors potentially associated with neighbourhood self-selection (choosing to live in neighbourhoods with specific characteristics) and MVPA/ST (see Fig. S1 and Supplementary information on Methods in Additional file 1). Residential density was included as a confounder of other environment-outcome relationships because it impacts on public transport and the availability of various destinations (Fig. 1) [48]. Study site and physical function (SPPB) were examined as moderators of environment-MVPA/ST associations by adding interaction terms to the main effect models. Significant moderation effects were probed by estimating associations for each study site and/or at three values of SPPB (mean and 1 standard deviation below and above the mean).

Based on previous studies, it was hypothesised that, in the whole sample, neighbourhood residential density would be unrelated to MVPA [9] but positively related to ST [49] (Fig. 1). MVPA was expected to be positively, and ST negatively, related to the densities of commercial and civic destinations, recreation facilities, public transport stops and number of parks in the neighbourhood [9, 11, 49]. SPPB was hypothesised to act as a moderator of the associations between density of commercial and civic destinations, recreation facilities, parks and MVPA [12, 21]. Specifically, stronger positive associations between these characteristics and MVPA were expected in participants with worse physical function. No hypotheses were formulated regarding SPPB as a moderator of environment-ST associations and study site as a moderator of environment-MVPA/ST associations due to lack of findings in the literature.

#### Mediation analyses of the effects of neighbourhood environmental attributes on MVPA and ST

Mediation effects were examined using the joint-significance test [50] and following the steps detailed in the Additional file 1. According to the joint-significance test, mediation is confirmed if the associations (regression coefficients) between an exposure and its mediator(s), and the exposure-adjusted associations between the mediator(s) and the outcome are statistically significant. The moderating role of study site was examined across all pathways linking environmental attributes with MVPA/ST, whilst the moderating role of SPPB was considered only for the direct effects of the environment on car ownership, MVPA and ST, and for the effects of car ownership on MVPA and ST. All analyses were conducted in R version 3.4.3 [51] using the packages ‘mgcv’ version 1.8.22 [46] and ‘multcomp’ version 1.4.8 [52]. Further details about the analyses are reported in the Additional file 1.

It was hypothesised that higher residential density would be positively related to public transport and various destination densities (Fig. 1) [48]. Unpublished data from a previous study conducted in Hong Kong suggested that these relationships might be curvilinear (inverted-U or concave down, increasing). Household car ownership was expected to be more prevalent among respondents living in areas with lower residential, commercial/civic destination, recreation and public transport densities [53]. We hypothesised that MVPA would be unrelated to residential density [9, 12] and positively associated with commercial and civic destinations, recreation facilities, public transport stops and number of parks [9]. No directional hypotheses were formulated about the main effects of car ownership on MVPA because of divergent findings in the literature [54–56]. Residential density was expected to be positively related [49], and other environmental attributes [11, 49] and MVPA [57] to be negatively related, to ST. No directional hypotheses were formulated about the direct effects of car ownership on ST because of divergent findings in the literature [55]. SPPB was expected to moderate the associations of car ownership [55, 58] with MVPA and ST (car ownership related to more MVPA and less ST in those with lower physical function), and public commercial/civic destinations, parks and recreation facilities with MVPA [12, 21] as explained above. No hypotheses were formulated regarding differences in effects across study sites.

## Results

On average, participants accumulated 56 and 414 daily minutes of MVPA and ST, respectively, with Hong Kong older adults showing higher levels of activity than their Belgian counterparts (Table 1). The Belgian sample was more educated and had a markedly higher prevalence of household car ownership. Hong Kong neighbourhoods had higher residential and recreation densities than Ghent.

### Total effects of neighbourhood environment attributes on MVPA and ST

Table 2 reports the total effects of neighbourhood environmental attributes on MVPA and ST. MVPA was positively associated with commercial/civic destination density and number of parks, whilst ST was negatively associated with public transport density within 1 km from home. SPPB moderated the associations between residential density and MVPA, whereby residential density was positively associated with MVPA only in older adults with below-average physical function (Table 3). Positive associations of commercial/civic destination and recreation densities with MVPA and of residential density with ST were only observed in Belgian older adults (Table 3).

**Table 2:** Total effects (associations) of neighbourhood environmental attributes on accelerometer-assessed daily minutes of moderate-to-vigorous physical activity (MVPA) and sedentary time (ST)

		MVPA (min/day)		ST (min/day)	
Environmental attribute	Buffer size	e^*b*^ (95% CI)	*p*	*b* (95% CI)	*p*
Residential density (1000 dwellings/km^2^)	400 m	1.001^a^ (0.998, 1.004)	.455	−0.07 (− 0.43, 0.28)	.688
	1 km	1.002^a^ (0.999, 1.006)	.227	−0.18^b^ (− 0.62, 0.26)	.418
Commercial & civic destination density (10 destinations/km^2^)	400 m	1.004^b^ (0.999, 1.008)	.098	−0.21 (− 0.65, 0.41)	.651
	1 km	**1.009** **(1.003, 1.015)**	**.005**	−0.17 (−1.31, 0.96)	.764
Public transport density (stops/km^2^)	400 m	0.999 (0.997, 1.002)	.705	0.26 (−0.11, 0.64)	.172
	1 km	1.002 (0.995, 1.009)	.626	**−1.13** **(−2.16, −0.06)**	**.048**
Recreation density (facilities/km^2^)	400 m	1.002 (0.999, 1.005)	.259	0.03 (−0.37, 0.43)	.889
	1 km	1.004^b^ (0.999, 1.009)	.117	−0.13 (− 0.74, 0.48)	.676
Park number (parks in buffer)	400 m	0.991 (0.961, 1.023)	.594	1.71 (−2.33, 5.76)	.406
	1 km	**1.017** **(1.006, 1.028)**	**.003**	0.21 (−1.47, 1.88)	.809

e^*b*^, exponentiated regression coefficient, *CI* confidence intervals, *b* regression coefficient, *p* p-value. Models were adjusted for covariates listed in Table S1 (model T). ^a^ moderated by physical function. ^b^ moderated by study site

**Table 3:** Significant moderators of total effects (associations) of neighbourhood environmental attributes on accelerometer-assessed MVPA and ST

Interaction effect	e^*b*^	95% CI	*p*
***Outcome: MVPA***			
Residential density – 400 m buffer (1000 dwellings/km^2^) by SPPB	0.999	0.998, 1.000	.015
Association at mean – 1 SD value of SPPB (8.08 points)	1.004	1.000, 1.007	.048
Association at mean value of SPPB (10.02 points)	1.001	0.998, 1.004	.531
Association at mean + 1 SD value of SPPB (11.96 points)	0.998	0.995, 1.002	.370
Residential density – 1 km buffer (1000 dwellings/km^2^) by SPPB	0.998	0.997, 0.999	.003
Association at mean – 1 SD value of SPPB (8.08 points)	1.006	1.002, 1.010	.009
Association at mean value of SPPB (10.02 points)	1.002	0.999, 1.006	.219
Association at mean + 1 SD value of SPPB (11.96 points)	0.999	0.994, 1.003	.582
Commercial & civic destination density – 400 m buffer (10 destinations/km^2^) by Study site	1.010	1.002, 1.018	.017
Association in Hong Kong	0.998	0.992, 1.005	.613
Association in Ghent	1.008	1.003, 1.014	.004
Recreation density (facilities/km^2^) – 1 km buffer by Study site	1.027	1.004, 1.050	.023
Association in Hong Kong	1.002	0.997, 1.007	.326
Association in Ghent	1.029	1.007, 1.052	.010
***Outcome: sedentary time (average min/day)***	***b***	**95% CI**	***p***
Residential density – 1 km buffer (1000 dwellings/km^2^) by Study site	2.52	0.14, 4.90	.038
Association in Hong Kong	0.00	−0.50, 0.50	.953
Association in Ghent	2.52	0.05, 4.99	.046

e^*b*^ exponentiated regression coefficient, *CI* confidence intervals, *b* regression coefficient, *p* p-value, *SPPB* Short Physical Performance Battery, 400 m and 1 km buffers denote street-network residential buffer sizes. Models were adjusted for covariates listed in Table S1 (model T)

### Direct and mediated effects of neighbourhood environmental attributes on MVPA and ST

**Fig. 2:**
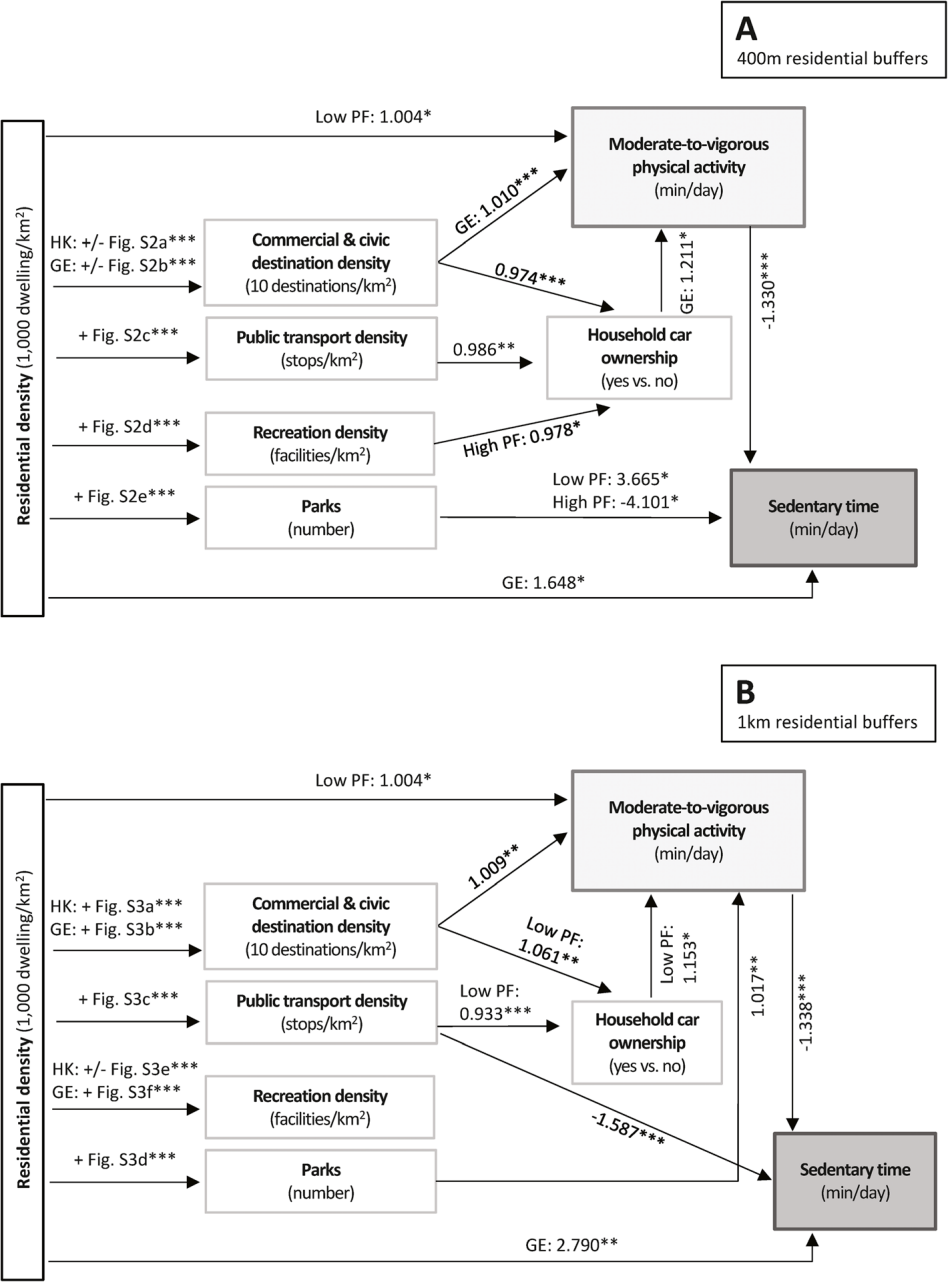
Direct and indirect effects of neighbourhood environmental attributes on accelerometer-assessed moderate-to-vigorous physical activity and sedentary time. Panels **a** and **b** report the results for 400 m and 1 km street-network residential buffers, respectively. Only significant associations (*p* < .05) are reported. Significant curvilinear associations between pairs of variables are labelled by their direction (+, positive; −, negative; +/−, positive and negative) and the figure number depicting them (e.g., Fig. S2a referring to Fig. S2 - panel A, representing the association between neighbourhood residential density and commercial and civic destination density within 400 m residential buffers). Values next to arrows pointing at ‘Household car ownership’, ‘Moderate-to-vigorous physical activity’ and ‘Sedentary time’ represent odds ratios, exponentiated regression coefficients and regression coefficients, respectively. Detailed results, point estimates and confidence intervals of all examined associations are reported in Tables S2-S5 and Figs. S2 and S3 included in the Additional file 1. * *p* < .05; ** *p* < .01; *** *p* < .001

Figure 2 summarises the findings of the mediation analyses. Detailed model outputs (point estimates, 95% CI and *p*-values for all regression coefficients) are presented in Tables S2-S5 (Additional file 1). Residential density within 400 m and 1 km residential buffers was found to have a direct positive effect on MVPA in older adults with below-average physical function (e^*b*^ for 400 m = 1.004; 95% CI: 1.000, 1.008; *p* = .038; e^*b*^ for 1 km = 1.004; 95% CI: 1.000, 1.009; *p* = .049) and on ST in older adults from Ghent (*b* for 400 m = 1.65; 95% CI: 0.25, 3.05; *p* = .021; *b* for 1 km = 2.79; 95% CI: 0.71, 4.87; *p* = .009). The indirect effects of residential density on MVPA and ST through other environmental attributes and household car ownership were mixed. For example, 400 m-buffer residential density showed a negative indirect effect on MVPA in Belgian older adults by being positively associated with public transport and recreation densities that, in turn, were negatively related to the odds of having a car in the household in all or only older adults with above-average physical function, and not having a car in the household was, in turn, predictive of less MVPA in Belgian older adults (Fig. 2 – panel A). In contrast, the indirect effects of 400 m-buffer residential density on MVPA via commercial/civic destination density and household car ownership were curvilinear due to the curvilinear relationship between the two neighbourhood attributes (Fig. S2 – panels A and B). Specifically, an increase in residential density up to 15,000 dwellings/km^2^ in Ghent was associated with an increase in the number of commercial/civic destinations which, after controlling for household car ownership, was positively associated with MVPA in Belgian older adults. However, further increases in residential density were associated with a decline in number of commercial/civic destinations (Fig. S2 – panel B), resulting in lower MVPA in Belgian older adults (Fig. 2 – panel A). Whilst 400 m-buffer commercial/civic destination density showed a positive direct effect on MVPA in the Belgian sample, its indirect effect mediated by household car ownership was negative since having more commercial/civic destinations was negatively associated with having a car and the latter was positively related to MVPA in Belgian older adults.

The indirect effects of 1 km-buffer residential density and direct effects of other 1 km-buffer environmental attributes on MVPA did not depend on study site (Fig. 2 – panel B). The indirect effects of residential density on MVPA mediated by commercial/civic destination density and number of parks were positive. Household car ownership was a mediator of the associations of commercial/civic destination and public transport stops/stations density with MVPA. These two environmental attributes were, respectively, positively and negatively related to having a car in the household in those with below-average physical function, and household car ownership was positively related to MVPA in the same subgroup of older adults.

As MVPA was negatively related to ST, all environmental attributes affecting MVPA also had an indirect effect on ST (through MVPA) but in the opposite direction (Fig. 2 – panels A and B). In addition to these pathways, a direct effect of 400 m-buffer number of parks on ST moderated by physical function was observed. Older adults with below-average physical function accumulated more ST if they had a larger number of parks in their 400 m residential buffer, whilst the opposite held true for those with above-average physical function (Fig. 2 – panel A). Additionally, 1 km-buffer public transport density showed a negative direct effect on ST (Fig. 2 – panel B).

## Discussion

Whilst a relatively large number of studies have examined objective neighbourhood environmental correlates of older adults’ PA, only a couple focused on accelerometer-assessed MVPA defined using cut-points appropriate for this age group [9]. There is also a scarcity of studies on environmental correlates of older adults’ accelerometer-assessed ST and on the moderating role of physical function. We addressed these knowledge gaps by pooling relevant comparable data collected in two culturally diverse populations living in two cities with remarkably different levels of residential density and car ownership, namely Hong Kong (China) and Ghent (Belgium).

Commercial/civic destination density and number of parks within 1 km from home were the only environmental attributes with significant positive total and direct effects on MVPA that were consistent across cities and older adults’ physical function levels. A similar finding was observed for public transport density within 1 km from home with respect to ST. In contrast, the total and direct effects of residential density on MVPA depended on physical function, and those of residential density on ST depended on study site. Study site was also a moderator of the total effects of 1 km-buffer recreation density and 400 m-buffer commercial/civic destination density on MVPA. The analysis of direct and indirect effects of residential density and other environmental attributes on MVPA and ST revealed a complex network of potential inconsistent pathways of influence linking all environmental attributes to MVPA and ST in the whole sample or in subgroups of participants (e.g., Belgian older adults or older adults with below-average physical function).

### Neighbourhood environment and MVPA

Availability and access to commercial destinations and services is one of the neighbourhood environmental features most consistently associated with PA in older adults [8, 9, 59]. Having access to shops, food outlets and other services promotes walking for transport [8], which has been found to contribute substantially to the accumulation of PA in older adults [38, 60, 61]. Thus, it is perhaps not surprising that, in the present study, commercial/civic destination density was equally positively associated with MVPA in Hong Kong as well as Belgian older adults irrespective of their level of physical function. Whilst, in essence, this finding is not new, it is noteworthy that the present study accounted for the potential confounding effects of residential density, while previous studies did not [12, 62, 63]. It is important to adjust for residential density because increases in population density do not only lead to increases in amenities. They also provide more opportunities for social contacts. With a larger social network, older adults are more likely to accrue more MVPA by walking to/from other individuals’ homes, which are among the most frequently reported walking destinations in this age group [64, 65]. It is interesting that whilst a positive association between 1 km-buffer commercial/civic destination density and MVPA was observed in both cities, this was not the case for the same environmental indicator based on 400 m-radius residential buffers. Sufficient evidence for a positive association was found in the Belgian sample only. This may be in part due to Hong Kong older adults being more physically active (with 60% more MVPA than Belgian older adults in this study) and generally accumulating greater volumes of transportation walking than their Belgian counterparts [21, 38, 66, 67] which, in turn, would make them more willing to walk further than 400 m from home for errands. Studies with data on older adults’ mobility behaviour and activity locations would be needed to test these hypotheses.

Access to parks and recreation facilities are neighbourhood environmental features that have been previously linked to higher levels of total PA [9], transportation walking [8] and leisure-time PA [10] among older adults. In line with these findings, we observed a positive association between the number of parks within 1 km from home and MVPA in both cities, irrespective of the level of physical function. In contrast, recreation density was positively related to MVPA only in Belgian older adults. An earlier study on Hong Kong older adults had found a positive association between recreation density and accelerometer-assessed MVPA [12]. However, that study used a much higher accelerometer cut-point to identify time spent in MVPA which might have captured vigorous PA (e.g., exercising in a gym) and missed a large proportion of moderate-intensity activities, such as walking. This would have yielded an inflated association between recreation density and MVPA. Though a high proportion of Hong Kong older adults engage in leisure-time PA other than walking [68], these activities are not particularly vigorous [68] and are likely performed in parks, sport fields and free-of-charge community and senior centres rather than commercial gyms and recreation/sport centres [69]. This may be one of the reasons why this study did not find an association between recreation density and MVPA in Hong Kong older adults.

Recent systematic reviews reported strong evidence of a positive association between access/availability of public transport and self-reported PA [9], transportation walking [9] and leisure-time walking within the neighbourhood [10]. In contrast, this study failed to observe a significant total main effect of public transport density on accelerometer-assessed MVPA. In line with our findings, Barnett and colleagues [9] also reported no evidence of an association between public transport and objectively-assessed PA. Although having good access to affordable public transport may promote walking to/from public transport stops and walking outside the neighbourhood, especially among older adults without a car, it may also reduce the amount of walking to/from other destinations in the neighbourhood [70] and, thus, yield no gain in PA. Studies assessing activity locations via GPS units, smartphones or web-assisted interviews would be required to verify the validity of these hypotheses.

The present study also found negative indirect effects of public transport density on MVPA mediated by household car ownership. Specifically, as expected, older adults living in a neighbourhood with high public transport density were less likely to own a car [70]. However, not having a car in the household was associated with less rather than more MVPA in Belgian older adults and those with below-average physical function. Similarly, two studies in the UK reported higher levels of objectively-assessed PA in older drivers vs. non-drivers [55, 71], which has been attributed to cars providing the extra support that older adults with mobility or health problems need to maintain their active lifestyle and interaction with the community [71]. Yet, this supposition may apply only to locations where car ownership and driving is highly prevalent among older adults, such as Western countries.

Whilst, as hypothesised, no significant total main effect of residential density on MVPA was observed, positive total and direct effects were found in older adults with below-average physical function. As noted earlier, densely-populated neighbourhoods provide plentiful opportunities for social interaction, a greater variety of amenities and shorter distances between destinations than low-density neighbourhoods. These attributes are particularly important to mobility-impaired older adults who, although less likely to engage in recreational PA [72], may continue to walk for utilitarian purposes in their local community. Whilst a few other studies have also found stronger positive associations between overall access to services and PA in mobility-impaired vs. non-impaired older adults [21, 22], the overall results are mixed [9] warranting further investigation.

Apart from finding positive direct effects of residential density on MVPA in participants with lower physical function, this study uncovered several inconsistent indirect effects mediated by other environmental attributes and household car ownership. When considering environmental attributes within 400 m from home, residential density had a positive indirect effect on MVPA via commercial/civic destination density, and negative indirect effects via commercial/civic destination, public transport and recreation density through household car ownership in Belgian older adults. The negative indirect effects were due to various amenity densities being negatively related to the likelihood of having a car in the household and, as noted earlier, having a car in the household being associated with more MVPA in Belgian older adults. Whilst the positive association between car ownership and MVPA has been discussed earlier, here it is worth commenting on the possible reasons for observing a negative association between recreation density and car ownership only in older adults with above-average physical function. Recreational facilities are more relevant to healthy and mobile individuals able to participate in sports and exercise than to those with impaired physical function [22, 72]. Hence, easy access to recreational facilities from home may be a stronger motivator for not owning a car among fully mobile older adults, which is another hypothesis that needs to be evaluated in future studies.

The indirect effects of residential density within 1 km residential buffers on MVPA were more positive than those observed for the smaller residential buffers and extended to both cities (Fig. 2). Only one negative indirect effect was observed via public transport density and car ownership in mobility-impaired older adults. In this regard, it is unclear why commercial/civic destination density was associated with a higher likelihood of car ownership in those with mobility impairment. One reason could be the higher levels of traffic and crowdedness typically found in such locations which may deter active transportation in this group of older adults.

### Neighbourhood environment and ST

Although only public transport and residential densities showed significant total and direct effects on ST, one of which held true only for the Belgian sample, all environmental attributes affecting MVPA had also an indirect, but reverse, effect on ST. This is because a negative association was observed between MVPA and ST. The actual effects of neighbourhood characteristics deemed to provide opportunities for an active lifestyle on PA are usually small [24]. This is even more the case for ST as the effects of the environment may be, in this case, channelled through PA and, thus, even more distal (see Fig. 1). Under such a scenario and the presence of inconsistent mediating mechanisms, it is understandable that, despite the scarcity of significant total effects, a considerable number of indirect effects of environmental attributes on ST were found [73, 74]. These findings suggest that activity-friendly environments may help older adults replace ST with MVPA.

Negative total and direct effects of public transport density on ST were observed in both cities and across people with different levels of physical function. Public transport density is a measure of regional accessibility [75] particularly relevant to residents with amenity-poor neighbourhoods. It may promote short utilitarian walking trips which, in this age group, are often of light-to-moderate intensity [12] and, hence, may be reflected in lower levels of ST rather than higher levels of MVPA [12, 42]. Parks within 400 m from home also exerted a direct effect on ST, which was moderated by physical function. In contrast, as noted earlier, the number of parks within 1 km from home had a positive effect on MVPA. Older adults may leisurely stroll to/from parks close to home (i.e., within 400 m from home) and walk faster to/from parks further away from home to compensate for the time cost of their trip. After reaching their destination, mobility-impaired individuals may prefer spending their time sitting and socialising in the park, whilst their counterparts may prefer strolling around the park.

Finally, a total and direct positive effect of residential density on ST was observed only in Belgian older adults. This latter finding mirrors those of international studies in adults [76]. Differences in housing between Ghent and Hong Kong may be responsible for the observed difference in associations. High-density European urban areas are typified by multi-storey residential buildings with relatively small apartments, whilst low-density areas are characterised by larger detached houses where older adults have more opportunities to engage in gardening and housework and, hence, reduce the time they spend in sedentary activities. In contrast, in Hong Kong, apartments are typically small throughout the whole territory and detached houses with gardens are very rare and unaffordable to older adults.

### Limitations

Several limitations need to be considered in interpreting the findings of this study. First, this study is cross-sectional. Hence, evidence of causality to support policy changes and environmental interventions is limited. Second, the findings from these two cities may not be generalisable to other geographical locations, especially low-middle income countries [77]. Third, the sampling strategy was designed to maximise the variability of environmental attributes within the cities. Therefore, the samples may not be representative of the populations of older adults in the two cities. Fourth, eligibility criteria for participation in the study included the ability to walk without assistance for a short distance without major difficulties. Thus, the findings apply to older adults with moderate-to-high mobility levels. Fifth, the eligibility criterion related to the ability to walk without assistance differed across the two study sites. However, fortunately, the between-site difference in average SPPB scores was minimal (0.17 points). Sixth, data on activity locations was not collected. It would have been useful and informative to distinguish between MVPA/ST accumulated within vs. outside the 400 m and 1 km residential buffers to better understand the impact of the neighbourhood environment on MVPA/ST in older adults. However, collection of location data via Global Positioning System (GPS) devices is challenging in ultra-dense cities like Hong Kong [78]. Seventh, as the response rate in the two cities differed (71% vs 45%) the degree of sampling bias might have also differed and contributed to the observed moderating effects of study site. Finally, this study treated car ownership as a potential mediator of environment-MVPA/ST associations, while it could have also been considered as a factor influencing neighbourhood self-selection (i.e., a confounder). As older adults typically have lower income than their younger counterparts, they are less likely to afford living in their preferred type of neighbourhood. This is especially the case in Hong Kong [12]. Hence, in this study, it seemed more reasonable to consider car ownership as a mediator.

## Conclusions

As evidenced in this study, an analysis of potential pathways through which specific neighbourhood environmental attributes affect older adults’ PA and ST can reveal competing influences that remain concealed when considering only the total effects of the environment on PA and ST. The revelation of competing mechanisms is important for the development of effective environmental interventions. This study suggests that, in general, neighbourhood characteristics that support MVPA also reduce ST by helping older adults replace ST with MVPA. These neighbourhood characteristics are parks and commercial and civic destinations within 1 km from home. They were found to be equally relevant to Hong Kong and Belgian older adults with different levels of physical function. Public transport and recreational facilities appear to have a negative impact on MVPA by reducing the likelihood of car ownership, but only in subgroups of older adults. The fact that car ownership may be necessary to maintain a certain level of MVPA in some suggests that the affordability, frequency, comfort and intelligibility of the current public transport systems may be inadequate for an ageing population, as was recently noted in a qualitative study of Chinese older adults in Melbourne [79]. Yet, the present study also suggests that public transport may help older adults to reduce ST irrespective of geographical location and physical function level. Finally, this study also revealed an intricate network of contrasting linear and curvilinear pathways of influence of residential density on MVPA and ST, which partly depended on the geographical context and individual physical function. Urban densification can lead to positive and negative impacts on older adults’ levels of activity. By identifying and strengthening the context-specific pathways that support MVPA and minimising or mitigating those that discourage it, we can create cities that can sustainably support healthy and active ageing.

## Supplementary information

**Additional file 1: **Supplementary Information on Methods including (in order of appearance): **Figure S1.** Directed acyclic graph depicting the hypothesised relations between neighbourhood residential density, other environmental attributes, household car ownership, covariates and outcome variables (moderate-to-vigorous physical activity and sedentary time). Detailed description of statistical analyses (generalised additive mixed models and mediation analyses) and hypotheses. **Table S1.** Outline of regression analyses. Supplementary Results including (in order of appearance): Direct and mediated effects of neighbourhood environmental attributes on MVPA and ST. **Table S2.** Step 1: Direct effects of neighbourhood residential density on other environmental attributes [pathways 1 in Fig. 1]. **Figure S2.** Shape of significant nonlinear relationships of residential density with environment attributes (400 m street-network residential buffers). **Figure S3.** Shape of significant nonlinear relationships of residential density with environment attributes (1 km street-network residential buffers). **Table S3.** Step 2: Direct effects of neighbourhood environmental attributes on household car ownership (ref: no car) [pathways 2 in Fig. 1]. **Table S4.** Step 3: Direct effects of neighbourhood environmental attributes and household car ownership on MVPA [pathways 3 in Fig. 1]. **Table S5.** Step 4: Direct effects of neighbourhood environmental attributes, household car ownership and MVPA on sedentary time [pathways 3 in Fig. 1].

## Data Availability

Due to lack of consent to share data outside the team of investigators, individual data cannot be made accessible.

## Abbreviations

AIC: Akaike Information Criterion
ALECS: Active Lifestyle and the Environment in Chinese Seniors
BEPAS: Belgian Environmental Physical Activity Study
EHCs: Elderly Health Centres
GAMMs: Generalised Additive Mixed Models
GIS: Geographic Information Systems
GPS: Global Positioning System
LFE: low-frequency filter
METs: Metabolic Equivalents of Task
MVPA: moderate-to-vigorous physical activity
PA: physical activity
SD: standard deviation
SES: socio-economic status
SNR: street-network residential
SPPB: Short Physical Performance Battery
ST: sedentary time
TPUs: Tertiary Planning Units
VIF: variance inflation factor
