# Supplementary material for: Urban environments and objectively-assessed physical activity and sedentary time in older Belgian and Chinese community dwellers: potential pathways of influence and the moderating role of physical function
Source: Int J Behav Nutr Phys Act. 2020 Jun 9;17:73. doi: 10.1186/s12966-020-00979-8 (PMC7285720; doi:10.1186/s12966-020-00979-8)
Supplement: Supplementary file 1 — Additional file 1: Supplementary Information on Methods including (in order of appearance): Figure S1. Directed acyclic graph depicting the hypothesised relations between neighbourhood residential density, other environmental attributes, household car ownership, covariates and outcome variables (moderate-to-vigorous physical activity and sedentary time). Detailed description of statistical analyses (generalised additive mixed models and mediation analyses) and hypotheses. Table S1. Outline of regression analyses. Supplementary Results including (in order of appearance): Direct and mediated effects of neighbourhood environmental attributes on MVPA and ST. Table S2. Step 1: Direct effects of neighbourhood residential density on other environmental attributes [pathways 1 in Fig. 1]. Figure S2. Shape of significant nonlinear relationships of residential density with environment attributes (400 m street-network residential buffers). Figure S3. Shape of significant nonlinear relationships of residential density with environment attributes (1 km street-network residential buffers). Table S3. Step 2: Direct effects of neighbourhood environmental attributes on household car ownership (ref: no car) [pathways 2 in Fig. 1]. Table S4. Step 3: Direct effects of neighbourhood environmental attributes and household car ownership on MVPA [pathways 3 in Fig. 1]. Table S5. Step 4: Direct effects of neighbourhood environmental attributes, household car ownership and MVPA on sedentary time [pathways 3 in Fig. 1]. [file 12966_2020_979_MOESM1_ESM.docx]

**Supplementary Information on Methods**


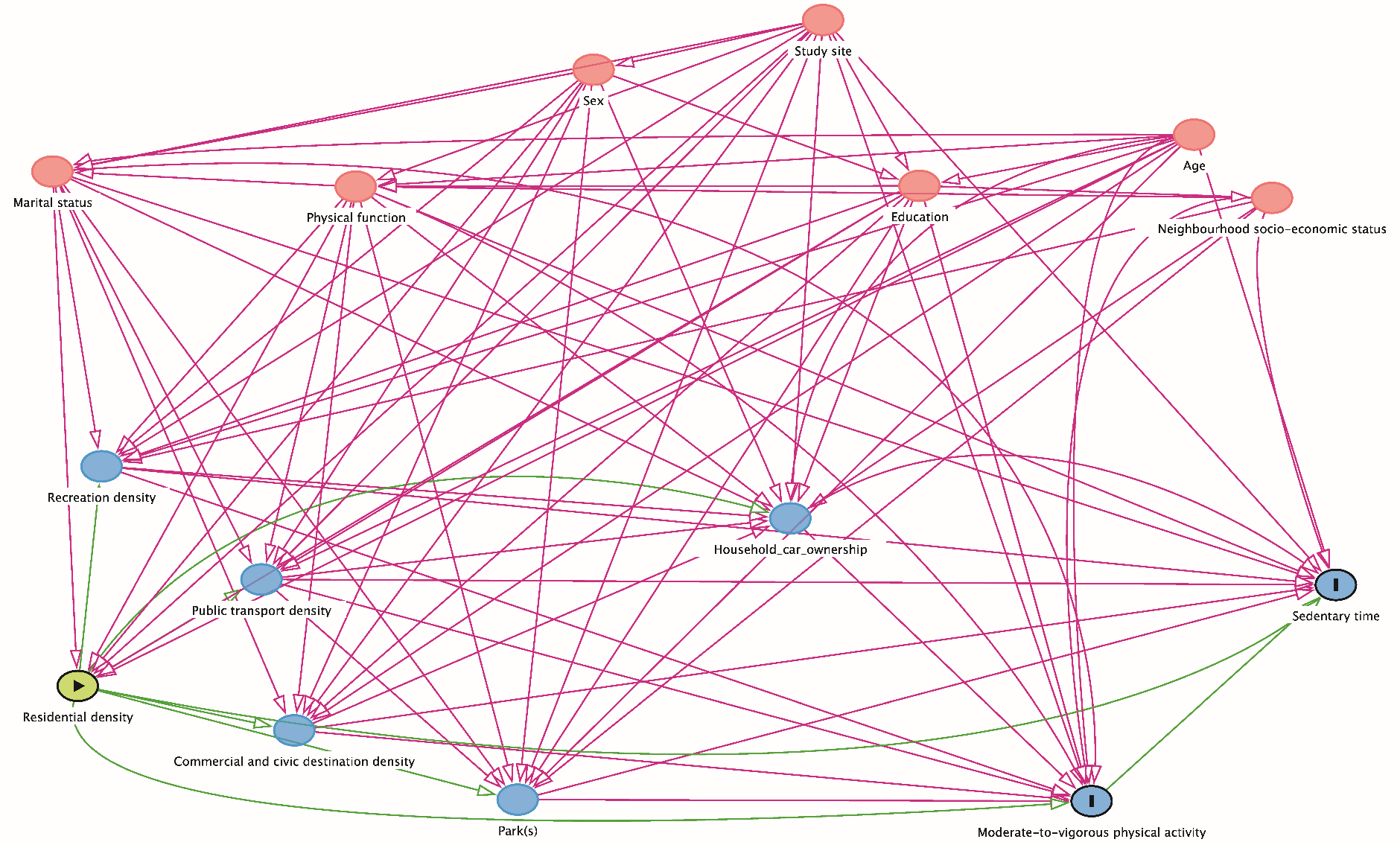
**Figure S1.** Directed acyclic graph (DAG) depicting the hypothesised relations between neighbourhood residential density (exposure, green oval), other environmental attributes, household car ownership (mediators, blue empty ovals), covariates (confounders, red ovals) and outcome variables (moderate-to-vigorous physical activity and sedentary time) (blue ovals with letter I). Through the DAG, we identified which covariates to include in the statistical analyses to sufficiently control for potential confounders. Biasing paths are in red, while causal paths are in green.

***Detailed description of statistical analyses (generalised additive mixed models and mediation analyses) and hypotheses***

1. *Total effects of neighbourhood environmental attributes on moderate-to-vigorous physical activity (MVPA) and sedentary time (ST)*

The confounder-adjusted total effects of neighbourhood environment characteristics on MVPA and ST and the moderating roles of SPPB and study sites were first estimated. Confounders (depicted in Figure S1) included factors potentially associated with neighbourhood self-selection (choosing to live in neighbourhoods with specific characteristics) and MVPA/ST. These were age (Aspvik et al., 2016; Schirmer et al., 2014; van Ballegooijen et al., 2019), sex (Aspvik et al., 2016; Lewis and Baldassare, 2010; van Ballegooijen et al., 2019), educational attainment (Lewis and Baldassare, 2010; van Ballegooijen et al., 2019), marital status (Espinel et al., 2015; Schirmer et al., 2014) and physical function represented by the total score on the SPPB (Espinel et al., 2015; James et al., 2015). Residential density was included as a confounder of other environment-outcome relationships because it impacts on public transport and the availability of various destinations (Figure 1 in main text) (Cervero and Kockelman, 1997). Finally, although the stratified sampling strategy adopted in this study controlled for the potential confounding effects of administrative unit-level socio-economic status (SES), administrative unit-level SES was included as a covariate to increase statistical power by accounting for unexplained outcome variance (Lingsma et al., 2010). In this regard, previous studies have found neighbourhood-level SES to be positively and negatively related to MVPA and ST, respectively (Hawkesworth et al., 2018; O’Donoghue et al., 2016).

Based on previous studies, it was hypothesised that, in the whole sample, neighbourhood residential density would be unrelated to MVPA (Barnett et al., 2017) but positively related to ST (Van Cauwenberg et al., 2014). MVPA was expected to be positively, and ST negatively, related to the densities of commercial and civic destinations, recreation facilities, public transport stops and number of parks in the neighbourhood (Barnett et al., 2015; Barnett et al., 2017; Van Cauwenberg et al., 2014). Study site and physical function (SPPB) were examined as moderators of the above associations by adding interaction terms to the main effect models. Significant moderation effects were probed by estimating associations for each study site and/or at three values of SPPB (mean and 1 standard deviation below and above the mean). SPPB was hypothesised to act as a moderator of the associations between density of commercial and civic destinations, recreation facilities, parks and MVPA (Barnett et al., 2016; Cerin et al., 2016). Specifically, stronger positive associations between these characteristics and MVPA were expected in participants with worse physical function. No hypotheses were formulated regarding SPPB as a moderator of environment-ST associations due to lack of findings in the literature.

1. *Mediation analyses of the effects of neighbourhood environmental attributes on MVPA and ST*

Mediation effects were examined using the joint-significance test (MacKinnon and Luecken, 2008). According to this test, mediation is confirmed if the associations (regression coefficients) between an exposure and its mediator(s), and the exposure-adjusted associations between the mediator(s) and the outcome are statistically significant. We first regressed densities of commercial and civic destinations, recreation facilities, public transport stops and number of parks (pathways 1 in Figure 1) onto residential density (Step 1 in Table S1). It was hypothesised that higher residential density would motivate increases in public transport and destination densities (Figure 1) (Cervero and Kockelman, 1997). Unpublished data from a previous study conducted in Hong Kong suggested that these relationships might be curvilinear (inverted-U or concave down, increasing). In step 2, we examined whether neighbourhood environmental attributes were related to household car ownership (pathways 2 in Figure 1) (Step 2 in Table S1). Household car ownership was expected to be more prevalent among respondents living in areas with lower residential, commercial/civic destination, recreation and public transport densities (Zhang et al., 2018).

In step 3, we estimated the direct and car ownership-mediated effects of environmental attributes on MVPA (pathways 3 in Figure 1; Step 3 in Table 1S). MVPA was expected to be unrelated to residential density (Barnett et al., 2017; Cerin et al., 2016) and positively associated with density of commercial and civic destinations, recreation facilities, public transport stops and number of parks (Barnett et al., 2017). No directional hypotheses were formulated about the main effects of car ownership on MVPA because of divergent findings in the literature (Ding et al., 2014; Hajna et al., 2019; Shoham et al., 2015). In step 4, we estimated the direct and MVPA- and car ownership-mediated effects of environmental attributes on ST. We hypothesised that residential density would be positively related (Van Cauwenberg et al., 2014), while other environmental attributes (Barnett et al., 2015; Van Cauwenberg et al., 2014) and MVPA (Garcia-Hermoso et al., 2015) would be negatively related to ST. No directional hypotheses were formulated about the direct effects of car ownership on ST because of divergent findings in the literature (Hajna et al., 2019).

Study site was examined as a moderator of all associations, while SPPB was examined as a moderator of environment-MVPA/ST, environment-car ownership and car ownership-MVPA/ST associations. Significant moderation effects were probed by estimating associations for each study site and/or associations at three values of SPPB (mean and 1 standard deviation below and above the mean). SPPB was expected to moderate the associations of car ownership (Amagasa et al., 2018; Hajna et al., 2019) with MVPA and ST (car ownership related to more MVPA and less ST in those with lower physical function), and public commercial/civic destinations, parks and recreation facilities with MVPA (Barnett et al., 2016; Cerin et al., 2016) as explained above. No hypotheses were formulated regarding differences in effects across study sites. All analyses were conducted in R version 3.4.3 (R Core Team, 2017) using the packages ‘mgcv’ version 1.8.22 (Wood, 2006) and ‘multcomp’ version 1.4.8 (Hothorn et al., 2008).

**Table S1. Outline of regression analyses**

| Step | Effect estimated | Covariates | Regression models |
| --- | --- | --- | --- |
| *Estimation of total effects of neighbourhood environmental attributes on moderate-to-vigorous physical activity (MVPA) and sedentary time (ST)* | |  |  |
| T*^#^ | Total effects of neighbourhood environmental attributes on MVPA and ST | Effect of residential density: Age, sex, marital status, educational attainment, neighbourhood-level SES, SPPB, study site, accelerometer wear time. Effect of other environmental attributes: as above + residential density | Two separate GAMMs for each outcome and buffer size (400m and 1km). GAMMs with Gamma variance and logarithmic link functions for MVPA. GAMMs with Gaussian variance and identity link functions for amount ST. |
| *Estimation of direct and mediated effects of neighbourhood environmental attributes on MVPA and ST* | |  |  |
| 1* | Direct effect of neighbourhood residential density on other environmental attributes [pathways 1 in Figure 1] | Age, sex, marital status, educational attainment, SPPB, study site | Four separate GAMMs, one for each environmental attribute other than residential density, by each buffer size (400m and 1km). GAMMs with Gamma or Negative Binomial variance and logarithmic link functions. |
| 2*^#^ | Direct effects of neighbourhood environmental attributes on household car ownership [pathways 2 in Figure 1] | Age, sex, marital status, educational attainment, neighbourhood-level SES, SPPB, study site | A GAMM with binomial variance and logarithmic link functions for each buffer size (400m and 1km). |
| 3*^#^ | Direct effects of neighbourhood environmental attributes and household car ownership on MVPA [pathways 3 in Figure 1] | Age, sex, marital status, educational attainment, neighbourhood-level SES, SPPB, study site, accelerometer wear time | A GAMM with Gamma variance and identity link functions for each buffer size (400m and 1km). |
| 4*^#^ | Direct effects of neighbourhood environmental attributes, household car ownership and MVPA on ST [pathways 4 in Figure 1] | Age, sex, marital status, educational attainment, neighbourhood-level SES, SPPB, study site, accelerometer wear time | A GAMM with Gaussian variance and identity link functions for each buffer size (400m and 1km). |

*Note:* SES, socio-economic status; GAMM, generalised additive mixed model; SPPB, Short Physical Performance Battery.

* The moderating effects of study site on were examined by adding two-way interaction terms to these models. ^#^ The moderating effects of physical function (total score on SPPB) were examined by adding two-way interaction terms to these models.

**Supplementary Results**

The Variance Inflation Factors (VIFs) for the variables included in the GAMMs ranged from 1.17 (marital status) to 3.84 (commercial and civic destination density), with mean and median values of 1.83 and 1.49, respectively. Given that all VIF values were substantially lower than 5 (Sheater, 2009), no multicollinearity issues were identified.

*Direct and mediated effects of neighbourhood environmental attributes on MVPA and ST*

Step 1 of the mediation analyses examined the associations of neighbourhood residential density with other environmental attributes (Table S1). Separate analyses were conducted for each buffer size. Table S2 and Figures S2 and S3 report the results of these analyses.

All associations between residential density and other environmental attributes were curvilinear and statistically significant (Table S2; Figure 2). Study site was a moderator of the associations between residential density (400m and 1km buffers) and commercial/civic destination density (400m buffer measures: Δ AIC = 188.6; 1km buffer measures: Δ AIC = 33.7), and between residential density (1km buffer) and recreation density (Δ AIC = 35.9). Hence, these associations were estimated for each site.

**Table S2. Step 1: Direct effects of neighbourhood residential density on other environmental attributes** [pathways 1 in Figure 1]

| Neighbourhood environmental attributes | Buffer size | *F*-ratio (degrees of freedom) | *p-*value |
| --- | --- | --- | --- |
| Commercial & civic destination density (destinations / km^2^) | 400m | Hong Kong:  *F* (4.92, 802.94) = 18.19; Figure S2 – panel A | <.001 |
|  |  | Ghent:  *F* (3.13, 802.94) = 77.32; Figure S2 – panel B | <.001 |
|  | 1km | Hong Kong:  *F* (2.96, 806.12) = 55.34; Figure S3 – panel A | <.001 |
|  |  | Ghent:  *F* (2.92, 806.12) = 100.84; Figure S3 – panel B | <.001 |
| Public transport density (stops / km^2^) | 400m | *F* (2.41, 808.59) = 11.69; Figure S2 – panel C | <.001 |
|  | 1km | *F* (2.92, 808.08) = 51.29; Figure S3 – panel C | <.001 |
| Recreation density (facilities / km^2^) | 400m | *F* (2.78, 808.22) = 6.53; Figure S2 – panel D | .001 |
|  | 1km | Hong Kong:  *F* (6.04, 803.97) = 18.44; Figure S3 – panel E | <.001 |
|  |  | Ghent:  *F* (1.00, 803.97) = 25.02; Figure S3 – panel F | <.001 |
| Parks (number in buffer) | 400m | *F* (2.62, 808.38) = 22.02; Figure S2 – panel E | <.001 |
|  | 1km | *F* (2.97, 808.03) = 139.70; Figure S3 – panel D |  |

*Note.* *F,* *F*-ratio of smooth term (curvilinear relationship). Generalised additive mix models with Gamma or negative binomial variance and logarithmic link functions. Random intercepts at the administrative unit level. Model adjusted for covariates listed in Table S1.

Figures S2 and S3 show the associations between neighbourhood residential density and other environmental attributes within 400m and 1km street-network residential buffers, respectively (step 1 of the mediation analyses). The relationship between 400m-buffer residential density and commercial/civic destination density was positive up to residential density values of ~13,000 dwellings/km^2^ in Hong Kong and up 15,000 dwellings/km^2^ in Ghent, then negative up to 30,000 dwellings/km^2^ in both cities and null thereafter (Figure S2, panels A and B). The remaining three environmental attributes increased with increases in residential density up 25,000-50,000 dwellings/km^2^ and then plateaued (Figure S2, panels C-E). Commercial/civic destination density based on 1km residential buffers increased with residential density up to ~32,000 dwellings/km^2^ in Hong Kong and up to ~15,000 dwellings/km^2^ in Ghent and then plateaued (Figure S3, panels A and B). A positive plateauing relationship was also observed between residential density and number of parks (Figure S3, panel D), while that between residential density and public transport density was positive and more linear (Figure S3, panel C). An inverted-U relationship between residential and recreation densities was observed in Hong Kong (Figure S3, panel E). In contrast, these two environmental attributes showed a positive linear relationship in Ghent (Figure S3, panel F).

Step 2 of the mediation analyses examined the direct effects of neighbourhood environmental attributes on household car ownership [pathways 2 in Figure 1]. Commercial/civic destination and public transport densities within 400m street-network distance from home were negatively related to the odds of having a car in the household (Table S3). Recreation density within 400m residential buffers was also negatively related to the odds of having a car in the household but only in those with above-average physical function. Among the 1km-buffer environmental measures, commercial/civic destination density was positively and transport density negatively associated with the odds of having a car in the household but only in those with below-average physical function (Table S3).


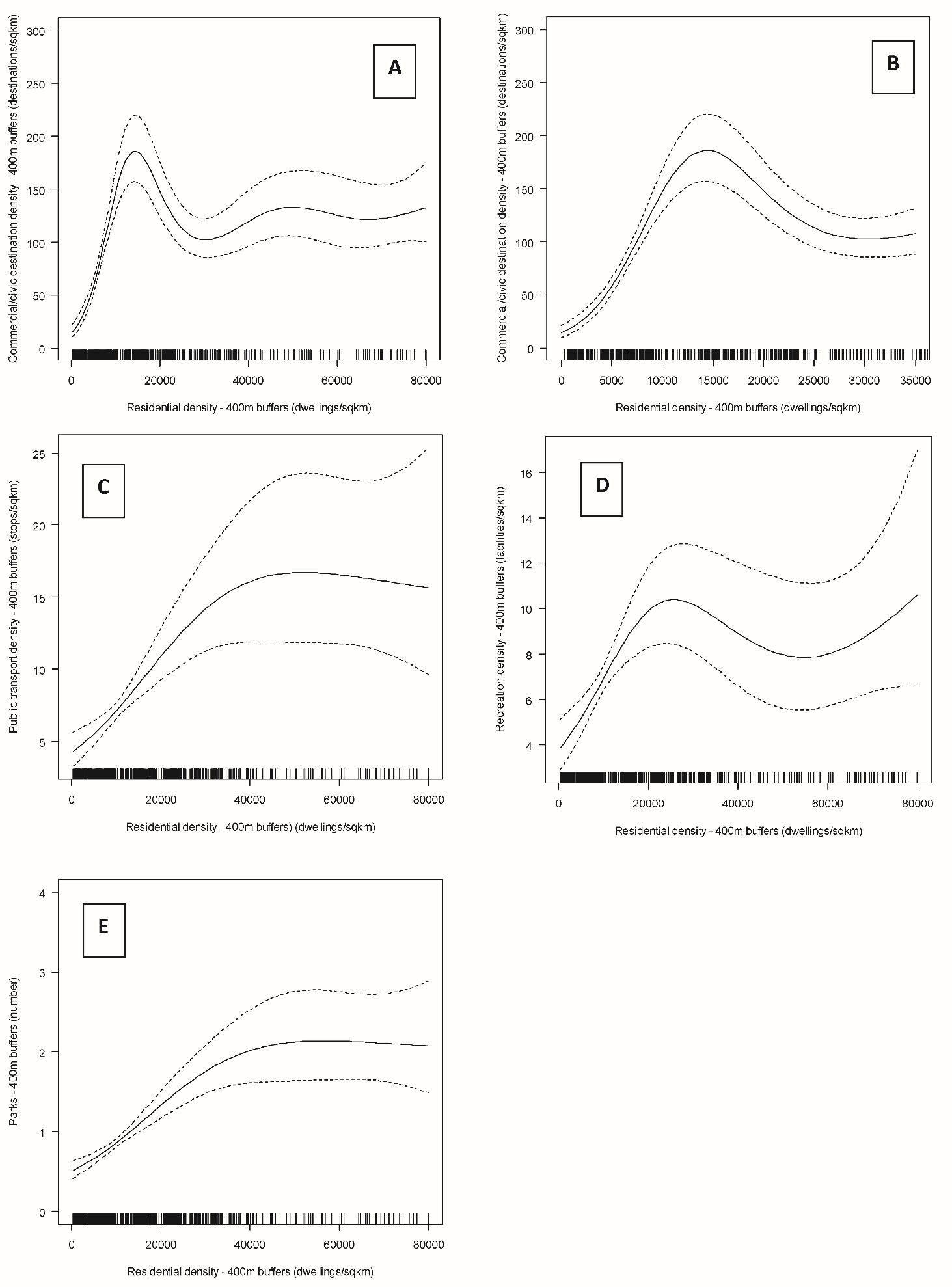


**Figure S2.** Shape of significant nonlinear relationships of residential density with environment attributes (400m street-network residential buffers): (A) commercial/civic destination density in Hong Kong; (B) commercial/civic destination density in Ghent; (C) public transport density; (D) recreation density; (E) number of parks. The solid lines represent point estimates and the dotted lines represent the 95% confidence intervals of the point estimates.

**
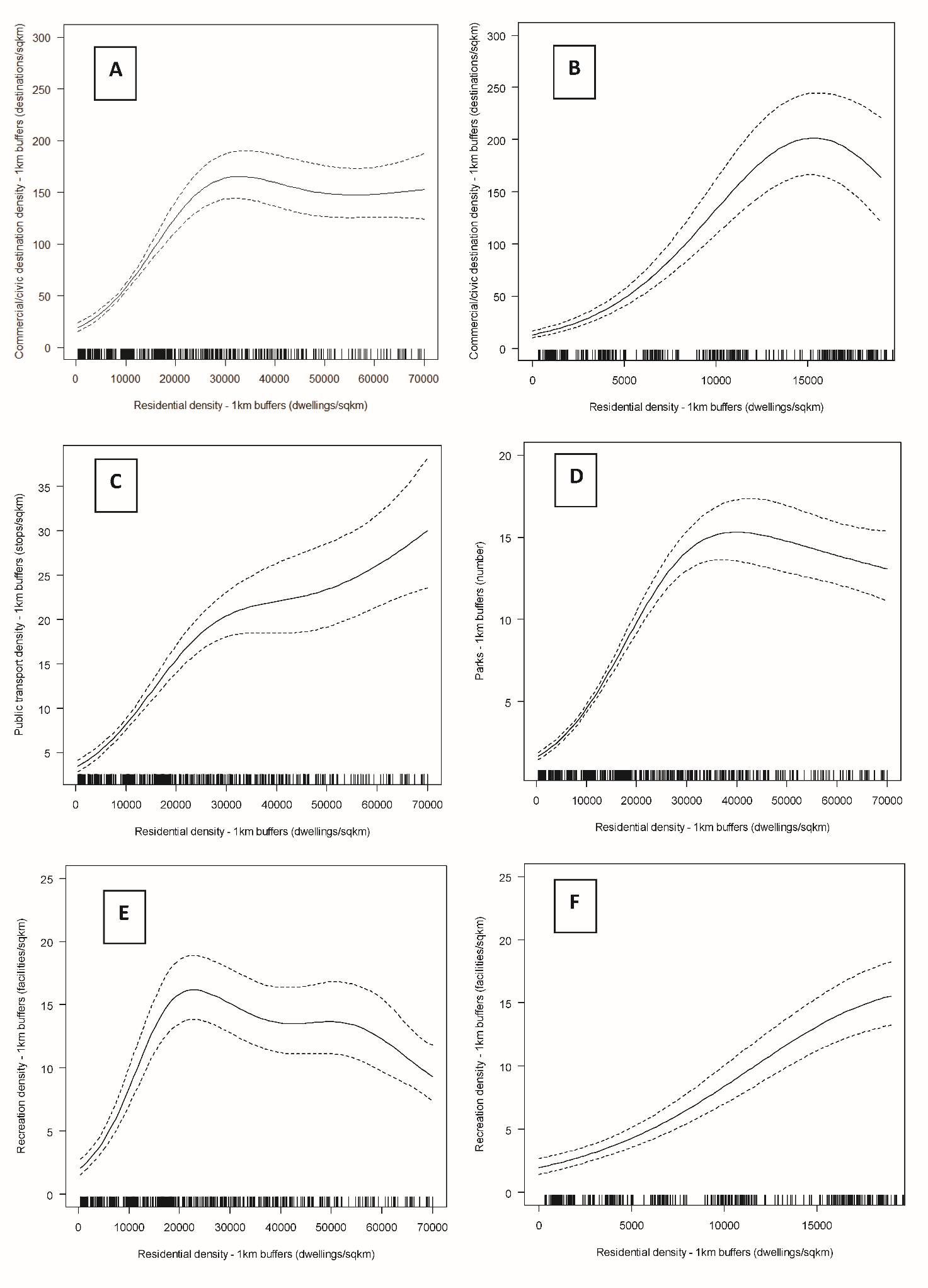
**

**Figure S3.** Shape of significant nonlinear relationships of residential density with environment attributes (1km street-network residential buffers): (A) commercial/civic destination density in Hong Kong; (B) commercial/civic destination density in Ghent; (C) public transport density; (D) number of parks; (E) recreation density in Hong Kong; (F) recreation density in Ghent. The solid lines represent point estimates and the dotted lines represent the 95% confidence intervals of the point estimates.

**Table S3. Step 2: Direct effects of neighbourhood environmental attributes on household car ownership** (ref: no car) [pathways 2 in Figure 1]

| Environmental attributes | OR (95% CI) | *p-*value |
| --- | --- | --- |
| Measures for 400m street-network residential buffers |  |  |
| Residential density (1,000 dwellings/km^2^) | 1.003 (0.991, 1.015) | .604 |
| Commercial & civic destination density (10 destinations / km^2^) | **0.974 (0.958, 0.989)** | **<.001** |
| Public transport density (stops / km^2^) | **0.921 (0.974, 0.997)** | **.002** |
| Recreation density (facilities / km^2^) by SPPB interaction | **0.994 (0.989, 0.999)** | **.024** |
| Association at mean – 1 SD value of SPPB (8.08 points) | 1.000 (0.986, 1.015) | .967 |
| Association at mean value of SPPB (10.02 points) | 0.989 (0.975, 1.003) | .110 |
| Association at mean + 1 SD value of SPPB (11.96 points) | **0.978 (0.959, 0.996)** | **.018** |
| Parks (number in buffer) | 0.921 (0.816, 1.039) | .182 |
| Measures for 1km street-network residential buffers |  |  |
| Residential density (1,000 dwellings/km^2^) | 0.993 (0.978, 1.008) | .326 |
| Commercial & civic destination density (10 destinations / km^2^) by SPPB interaction | **0.983 (0.971, 0.995)** | **.007** |
| Association at mean – 1 SD value of SPPB (8.08 points) | **1.061 (1.016, 1.108)** | **.008** |
| Association at mean value of SPPB (10.02 points) | 1.026 (0.992, 1.062) | .141 |
| Association at mean + 1 SD value of SPPB (11.96 points) | 0.992 (0.953, 1.033) | .702 |
| Public transport density (stops / km^2^) by SPPB interaction | **1.020 (1.008, 1.033)** | **.002** |
| Association at mean – 1 SD value of SPPB (8.08 points) | **0.933 (0.987, 0.970)** | **<.001** |
| Association at mean value of SPPB (10.02 points) | 0.970 (0.939, 1.003) | .072 |
| Association at mean + 1 SD value of SPPB (11.96 points) | 1.009 (0.968, 1.053) | .660 |
| Recreation density (facilities / km^2^) | 0.988 (0.970, 1.006) | .192 |
| Parks (number in buffer) | 0.956 (0.906, 1.008) | .099 |

*Note*. ref, reference category; OR, odds ratio; CI, confidence intervals; SPPB, Short Physical Performance Battery; SD, standard deviation. Generalised additive mix model with binomial variance and logit link functions. Random intercepts at the administrative unit level. Model adjusted for covariates listed in Table S1.

Step 3 of the mediation analyses examined the direct effects of neighbourhood environmental attributes and household car ownership on MVPA [pathways 3 in Figure 1]. A direct positive effect of 400m-buffer residential density on MVPA was observed in those with below-average physical function (Table S4). Older adults living in Ghent also showed a positive association between MVPA and commercial/civic destination density within 400m street-network distance from home. After adjusting for 400m-buffer environmental attributes, having a car in the household was associated with more MVPA in participants from Ghent only (Table S4). Among 1km-buffer environmental measures, residential density was positively associated with MVPA in those with below-average physical function, while commercial/civic destination density and number of parks were positively associated with MVPA irrespective of the level of physical function and study site (Table S4). After adjustment for 1km-buffer environmental attributes, household car ownership was predictive of a higher level of MVPA only in those with below-average physical function.

**Table S4. Step 3: Direct effects of neighbourhood environmental attributes and household car ownership on MVPA** [pathways 3 in Figure 1]

| Predictors | e*^b^* (95% CI) | *p-*value |
| --- | --- | --- |
| Measures for 400m street-network residential buffers |  |  |
| Residential density (1,000 dwellings/km^2^) by SPPB interaction | **0.999 (0.998, 1.000)** | **.026** |
| Association at mean – 1 SD value of SPPB (8.08 points) | **1.004 (1.000, 1.008)** | **.038** |
| Association at mean value of SPPB (10.02 points) | 1.001 (0.998, 1.004) | .366 |
| Association at mean + 1 SD value of SPPB (11.96 points) | 0.999 (0.995, 1.003) | .601 |
| Commercial & civic destination density (10 destinations / km^2^) by Study site interaction | **1.011 (1.003, 1.019)** | **.009** |
| Association in Hong Kong | 0.999 (0.993, 1.006) | .874 |
| Association in Ghent | **1.010 (1.004, 1.016)** | **<.001** |
| Public transport density (stops / km^2^) | 0.998 (0.995, 1.001) | .264 |
| Recreation density (facilities / km^2^) | 1.001 (0.998, 1.004) | .390 |
| Parks (number in buffer) | 0.977 (0.945, 1.010) | .164 |
| Household car ownership (ref: no car) by Study site interaction | **1.259 (1.014, 1.563)** | **.037** |
| Association in Hong Kong | 0.963 (0.841, 1.102) | .581 |
| Association in Ghent | **1.212 (1.027, 1.430)** | **.023** |
| Measures for 1km street-network residential buffers |  |  |
| Residential density (1,000 dwellings/km^2^) by SPPB interaction | **0.998 (0.997, 0.999)** | **.001** |
| Association at mean – 1 SD value of SPPB (8.08 points) | **1.004 (1.000, 1.009)** | **.049** |
| Association at mean value of SPPB (10.02 points) | 1.000 (0.996, 1.004) | .959 |
| Association at mean + 1 SD value of SPPB (11.96 points) | 0.996 (0.991, 1.000) | .076 |
| Commercial & civic destination density (10 destinations / km^2^) | **1.009 (1.003, 1.015)** | **.005** |
| Public transport density (stops / km^2^) | 0.993 (0.984, 1.001) | .104 |
| Recreation density (facilities / km^2^) | 1.001 (0.995, 1.006) | .829 |
| Parks (number in buffer) | **1.017 (1.005, 1.028)** | **.004** |
| Household car ownership (ref: no car) by SPPB interaction | **0.954 (0.909, 0.999)** | **.047** |
| Association at mean – 1 SD value of SPPB (8.08 points) | **1.153 (1.010, 1.316)** | **.035** |
| Association at mean value of SPPB (10.02 points) | 1.051 (0.950, 1.166) | .350 |
| Association at mean + 1 SD value of SPPB (11.96 points) | 0.954 (0.909, 0.999) | .629 |

*Note.* e*^b^*, exponentiated regression coefficient; CI, confidence intervals; SPPB, Short Physical Performance Battery; SD, standard deviation. Generalised additive mix models with Gamma variance and logarithmic link functions. Random intercepts at the administrative unit level. Model adjusted for covariates listed in Table S1.

Step 4 of the mediation analyses examined the direct effects of neighbourhood environmental attributes, household car ownership and MVPA on ST [pathways 4 in Figure 1]. Direct positive effects of residential density within 400m and 1km street-network distance from home on ST were observed only in older adults living in Ghent (Table S5). While a positive association between the number of parks within 400m residential buffers and ST was observed in those with below-average physical function, a negative association was observed in those with above-average physical function. Public transport density within 1km residential buffers was negatively associated with ST. Finally, after adjustment for 400m- and 1km-buffer environmental attributes and for household car ownership, strong negative associations between MVPA and ST was observed (Table S5).

**Table S5. Step 4: Direct effects of neighbourhood environmental attributes, household car ownership and MVPA on sedentary time** [pathways 3 in Figure 1]

| Predictors | e*^b^* (95% CI) | *p-*value |
| --- | --- | --- |
| Measures for 400m street-network residential buffers |  |  |
| Residential density (1,000 dwellings/km^2^) by SPPB interaction | **1.676 (0.256, 3.097)** | **.021** |
| Association in Hong Kong | -0.028 (-0.351, 0.295) | .865 |
| Association in Ghent | **1.648 (0.246, 3.045)** | **.021** |
| Commercial & civic destination density (10 destinations / km^2^) | 0.076 (-0.429, 0.580) | .768 |
| Public transport density (stops / km^2^) | -0.097 (-0.437, 0.244) | .577 |
| Recreation density (facilities / km^2^) | 0.100 (-0.251, 0.452) | .575 |
| Parks (number in buffer) by SPPB interaction | **-2.001 (-3.493, -0.509)** | **.009** |
| Association at mean – 1 SD value of SPPB (8.08 points) | **3.665 (0.420, 6.912)** | **.027** |
| Association at mean value of SPPB (10.02 points) | -0.218 (-3.755, 3.319) | .904 |
| Association at mean + 1 SD value of SPPB (11.96 points) | **-4.101 (-7.932, -0.271)** | **.036** |
| Household car ownership (ref: no car) | 0.411 (-1.178, 12.601) | .947 |
| MVPA (average min/day) | **-1.330 (-1.487, -1.173)** | **<.001** |
| Measures for 1km street-network residential buffers |  |  |
| Residential density (1,000 dwellings/km^2^) by Study site interaction | **2.855 (0.856, 4.854)** | **.005** |
| Association in Hong Kong | -0.065 (-0.488, 0.358) | .763 |
| Association in Ghent | **2.790 (0.712, 4.868)** | **.009** |
| Commercial & civic destination density (10 destinations / km^2^) | 0.250 (-0.702, 1.202) | .607 |
| Public transport density (stops / km^2^) | **-1.587 (-2.459, -0.713)** | **<.001** |
| Recreation density (facilities / km^2^) | -0.129 (-0.640, 0.381) | .619 |
| Parks (number in buffer) | 1.033 (-0.386, 2.451) | .153 |
| Household car ownership (ref: no car) | -0.757 (-12.930, 11.415) | .903 |
| MVPA (average min/day) | **-1.338 (-1.494, -1.182)** | **<.001** |

*Note.* *b*, regression coefficient; CI, confidence intervals; SPPB, Short Physical Performance Battery; SD, standard deviation; MVPA = moderate-to-vigorous physical activity. Generalised additive mix models with Gaussian variance and identity link functions. Random intercepts at the administrative unit level. Model adjusted for covariates listed in Table S1.

**References**

Amagasa S, Fukushima N, Kikuchi H, Takamiya T, Odagiri Y, Oka K, Inoue S. (2018). Drivers are more physically active than non-drivers in older adults. Int J Environ Res Public Health. 2018;15(6). pii: E1094. doi:

10.3390/ijerph15061094.

Aspvik NP, Viken H, Zisko N, Ingebrigtsen JE, Wisløff U, Stensvold D. (2016). Are older adults physically active enough – a matter of assessment method? The Generation 100 Study. PLoS ONE 11(11): e0167012. doi: 10.1371/journal.pone.0167012

Barnett A, Cerin E, Ching CS, Johnston JM, Lee RS. (2015). Neighbourhood environment, sitting time and motorised transport in older adults: a cross-sectional study in Hong Kong. BMJ Open. 5(4):e007557. doi: 10.1136/bmjopen-2014-007557.

Barnett A, Cerin E, Zhang CJP, Sit CHP, Johnston JM, Cheung MMC, Lee RSY. (2016). Associations between the neighbourhood environment characteristics and physical activity in older adults with specific types of chronic conditions: the ALECS cross-sectional study. Int J Behav Nutr Phys Act 13:53. doi:

10.1186/s12966-016-0377-7.

Barnett DW, Barnett A, Nathan A, Van Cauwenberg J, Cerin E; Council on Environment and Physical Activity (CEPA) – Older Adults working group. (2017). Built environmental correlates of older adults' total physical activity and walking: a systematic review and meta-analysis. Int J Behav Nutr Phys Act 14(1):103. doi: 10.1186/s12966-017-0558-z.

Cerin E, Zhang CJ, Barnett A, Sit CH, Cheung MM, Johnston JM, Lai PC, Lee RS. (2016). Associations of objectively-assessed neighborhood characteristics with older adults' total physical activity and sedentary time in an ultra-dense urban environment: Findings from the ALECS study. Health Place 42:1-10. doi: 10.1016/j.healthplace.2016.08.009.

Cervero R, Kockelman K. (1997). Travel demand and the 3DS: density, diversity, and design. Transport Res D – Tr E 2:199-219; doi: 10.1016/S1361-9209(97)00009-6.

Ding D, Sallis JF, Norman GJ, Frank LD, Saelens BE, Kerr J, Conway TL, Cain K, Hovell MF, Hofstetter CR, King AC. (2014). Neighborhood environment and physical activity among older adults: do the relationships differ by driving status? J Aging Phys Act 22(3):421-31. doi: 10.1123/japa.2012-0332.

Espinel PT, Chau JY, van der Ploeg HP, Merom D. (2015). Older adults' time in sedentary, light and moderate intensity activities and correlates: application of Australian Time Use Survey. J Sci Med Sport 18(2):161-6. doi: 10.1016/j.jsams.2014.02.012.

García-Hermoso A, Martínez-Vizcaíno V, Sánchez-López M, Recio-Rodriguez JI, Gómez-Marcos MA, García-Ortiz L; EVIDENT Group. (2015). Moderate-to-vigorous physical activity as a mediator between sedentary behavior and cardiometabolic risk in Spanish healthy adults: a mediation analysis. Int J Behav Nutr Phys Act 12:78. doi: 10.1186/s12966-015-0244-y.

Hajna S, White T, Panter J, Brage S, Wijndaele K, Woodcock J, Ogilvie D, Imamura F, Griffin SJ. (2019). Driving status, travel modes and accelerometer-assessed physical activity in younger, middle-aged and older adults: a prospective study of 90 810 UK Biobank participants. Int J Epidemiol 48(4):1175-1186.

doi: 10.1093/ije/dyz065.

Hawkesworth S, Silverwood RJ, Armstrong B, Pliakas T, Nanchalal K, Jefferis BJ, Sartini C, Amuzu AA, Wannamethee SG, Ramsay SE, Casas JP, Morris RW, Whincup PH, Lock K. (2018). Investigating associations between the built environment and physical activity among older people in 20 UK towns. J Epidemiol Community Health 72(2):121-131. doi: 10.1136/jech-2017-209440.

Hothorn T, Bretz F, Westfall P. (2008). Simultaneous inference in general parametric models. Biometrical J 50:346—363; doi: 10.1002/bimj.200810425.

James P, Hart JE, Arcaya MC, Feskanich D, Laden F, Subramanian SV. (2015). Neighborhood self-selection: the role of pre-move health factors on the built and socioeconomic environment. Int J Environ Res Public Health 12:12489-12504. doi: 10.3390/ijerph121012489.

Lewis PG, Baldassare M. (2010). The complexity of public attitudes toward compact development. J Am Planning Assoc 76:219-237; doi: 10.1080/01944361003646471.

Lingsma H, Roozenbeek B, Steyerberg E, IMPACT investigators. (2010). Covariate adjustment increases statistical power in randomized controlled trials. J Clin Epidemiol 63:1391; doi: 10.1016/j.jclinepi.2010.05.003.

MacKinnon DP, Luecken LJ. (2008). How and for whom? Mediation and moderation in health psychology. Health Psychol 27(2S):S99-S100. doi: 10.1037/0278-6133.27.2(Suppl.)S99.

O'Donoghue G, Perchoux C, Mensah K, Lakerveld J, van der Ploeg H, Bernaards C, Chastin SF, Simon C, O'Gorman D, Nazare JA; DEDIPAC Consortium. (2016). A systematic review of correlates of sedentary behaviour in adults aged 18-65 years: a socio-ecological approach. BMC Public Health 16:163. doi: 10.1186/s12889-016-2841-3.

R Core Team. (2017). R: A language and environment for statistical computing. Vienna, Austria: R Foundation for Statistical Computing. Available: https://www.R-project.org/.

Schirmer PM, van Eggermond MAB, Axhausen KW. (2014). The role of location in residential location choice models: a review of literature. J Transport Land Use 7:3-21; doi: 10.5198/jtlu.v7i2.740.

Sheather S. (2009). A Modern Approach to Regression with R. New York, NY: Springer.

Shoham DA, Dugas LR, Bovet P, Forrester TE, Lambert EV, Plange-Rhule J, Schoeller DA, Brage S, Ekelund U, Durazo-Arvizu RA, Cooper RS, Luke A. (2015). Association of car ownership and physical activity across the spectrum of human development: Modeling the Epidemiologic Transition Study (METS). BMC Public

Health 15:173. doi: 10.1186/s12889-015-1435-9.

van Ballegooijen AJ, van der Ploeg HP, Visser M. (2019). Daily sedentary time and physical activity as assessed by accelerometry and their correlates in older adults. Eur Rev Aging Phys Act 16:3. doi: 10.1186/s11556-019-0210-9.

Van Cauwenberg J, De Donder L, Clarys P, De Bourdeaudhuij I, Owen N, Dury S, De Witte N, Buffel T, Verté D, Deforche B. (2014). Relationships of individual, social, and physical environmental factors with older adults' television viewing time. J Aging Phys Act 22(4):508-17. doi: 10.1123/japa.2013-0015.

Wood SN. (2006). Generalised Additive Models: An Introduction with R, 2nd ed. Boca Raton, FL: Chapman & Hall/CRC.

Zhang Y, Lee C, Liu Q, Wu W. (2018). The socioeconomic characteristics, urban built environment and household car ownership in a rapidly growing city: evidence from Zhongshan, China. J Asian Archit Build 17:133-140. doi: 10.3130/jaabe.17.133.
